# The Exopolysaccharide of *Lactobacillus fermentum* UCO-979C Is Partially Involved in Its Immunomodulatory Effect and Its Ability to Improve the Resistance against *Helicobacter pylori* Infection

**DOI:** 10.3390/microorganisms8040479

**Published:** 2020-03-27

**Authors:** Valeria Garcia-Castillo, Guillermo Marcial, Leonardo Albarracín, Mikado Tomokiyo, Patricia Clua, Hideki Takahashi, Haruki Kitazawa, Apolinaria Garcia-Cancino, Julio Villena

**Affiliations:** 1 Laboratory of Bacterial Pathogenicity, Faculty of Biological Sciences, University of Concepcion, Concepcion Bio Bio 4030000, Chile; valeriagarcia@udec.cl; 2 Laboratory of Immunobiotechnology, Reference Centre for Lactobacilli (CERELA-CONICET), Tucuman CP4000, Argentina; gmarcial@cerela.org.ar (G.M.); lalbarracin@herrera.unt.edu.ar (L.A.); pclua@cerela.org.ar (P.C.); 3 Food and Feed Immunology Group, Graduate School of Agricultural Science, Tohoku University, Sendai 981-8555, Japan; mikado0403@gmail.com; 4 Laboratory of Computing Science. Faculty of Exact Sciences and Technology. Tucuman University, Tucuman CP4000, Argentina; 5 Laboratory of Plant Pathology, Graduate School of Agricultural Science, Tohoku University, Sendai 981-8555, Japan; hideki.takahashi.d5@tohoku.ac.jp; 6 Plant Immunology Unit, International Education and Research Centre for Food and Agricultural Immunology (CFAI), Graduate School of Agricultural Science, Tohoku University, Sendai 981-8555, Japan; 7 Livestock Immunology Unit, International Education and Research Center for Food and Agricultural Immunology (CFAI), Graduate School of Agricultural Science, Tohoku University, Sendai 981-8555, Japan

**Keywords:** *Lactobacillus fermentum* UCO-979C, exopolysaccharide, *H. pylori*, gastric cytokines, immunobiotics

## Abstract

*Lactobacillus fermentum* UCO-979C (Lf979C) beneficially modulates the cytokine response of gastric epithelial cells and macrophages after *Helicobacter pylori* infection in vitro. Nevertheless, no in vivo studies were performed with this strain to confirm its beneficial immunomodulatory effects. This work evaluated whether Lf979C improves protection against *H. pylori* infection in mice by modulating the innate immune response. In addition, we evaluated whether its exopolysaccharide (EPS) was involved in its beneficial effects. Lf979C significantly reduced TNF-α, IL-8, and MCP-1 and augmented IFN-γ and IL-10 in the gastric mucosa of *H. pylori*-infected mice. The differential cytokine profile induced by Lf979C in *H. pylori*-infected mice correlated with an improved reduction in the pathogen gastric colonization and protection against inflammatory damage. The purified EPS of Lf979C reduced IL-8 and enhanced IL-10 levels in the gastric mucosa of infected mice, while no effect was observed for IFN-γ. This work demonstrates for the first time the in vivo ability of Lf979C to increase resistance against *H. pylori* infection by modulating the gastric innate immune response. In addition, we advanced knowledge of the mechanisms involved in the beneficial effects of Lf979C by demonstrating that its EPS is partially responsible for its immunomodulatory effect.

## 1. Introduction

*Helicobacter pylori* (*H. pylori*) colonizes around 50% of the world population. This bacterium is able to dominate the gastric microbiota and lead to gastric inflammation (gastritis) in infected individuals [1,2]. The conventional treatment for *H. pylori* infection involves the combination of two antibiotics (amoxicillin/clarithromycin) and a proton pump inhibitor. Although this therapy has been useful in reducing the pathologies associated with *H. pylori*, its high cost, patient non-adherence to treatment, and the appearance of resistant strains, have led to the search for new innocuous, natural and healthier options, such as probiotics or plant derivatives [3,4,5].

In recent years, the scientific advances in probiotics have demonstrated their ability to improve resistance against infectious diseases including those produced by gastrointestinal, respiratory and urinary pathogens [6,7,8]. As probiotics have been proven to restore the microbiota balance, inhibit pathogen’s growth and/or modulate mucosal immune responses, these microorganisms have been widely used in the development of functional foods aiming to improve resistance against bacterial and viral pathogens [8,9,10]. For this reason, the research interest in the characterization of new probiotic strains with outstanding benefits on human health has been increased in recent years [11].

It is recognized that the gastric microbial community and the mucosal immune responses determine the outcome of *H. pylori* infection [12]. Alterations in the microbiota and deregulation of the inflammatory response during *H. pylori* infection can led to the development of important diseases such as peptic ulcer-, gastric cancer- and mucosa-associated lymphoid tissue (MALT) lymphoma [1,2]. Thus, probiotics have appeared as an alternative to modulate stomach microbiota and/or gastric inflammation and to reduce the severity and complications of *H. pylori* infection [13,14]. In this regard, our previous studies demonstrated the benefits of *Lactobacillus fermentum* UCO-979C against the pathogenic bacteria *H. pylori,* which were related to its capability of forming biofilms [15]. *L. fermentum* UCO-979C inhibited bacterial growth, reduced *H. pylori* urease activity, decreased pathogen adhesion to human gastric epithelial cells and beneficially regulated the inflammatory response in in vitro model by reducing the inflammatory chemokine IL-8 [16]. Besides, *L. fermentum* UCO-979C was able to differentially modulate the production of TNF-α, IFN-γ and IL-10 by gastric epithelial cells and macrophages, which may offer advantages in the protection against *H. pylori* infection by improving the clearance of the pathogen and the protection against inflammatory damage [17]. Futher in vitro studies demonstrated that the UCO-979C strain is a remarkable immunomodulatory agent due to its ability to differentially modulate the immune response triggered by Toll- like receptor 4 (TLR4) activation through the modulation of TLR negative regulators’ expression in epithelial cells [18].

Our previous studies with *L. fermentum* UCO-979C indicated the potential of this immunobiotic strain to beneficially modulate the immune response to *H. pylori* infection. However, the molecules of the UCO-979C strain involved in its immunomodulatory effect were not investigated in detail. Moreover, the ability of *L. fermentum* UCO-979C to modulate the immune response against the gastric infection in vivo has not been evaluated before. Therefore, the aim of this work was to evaluate the effect of *L. fermentum* UCO-979C or its secreted exopolysaccharide (EPS) on the immune response triggered by the pathogenic bacteria *H. pylori* in vitro and in vivo in a mouse model.

## 2. Materials and Methods

### 2.1. Microorganisms

*L. fermentum* UCO-979C [16] was obtained from the Bacterial Pathogenicity Laboratory culture collection at University of Concepción (Concepción, Chile). *L. fermentum* CRL973 was obtained from the CERELA-CONICET culture collection (Tucumán, Argentina). Lactobacilli strains were grown in Mann–Rogosa–Sharpe Agar (MRS) (Difco, Lawrence, KS, USA) at 37 °C. After an overnight growth, bacteria were transferred to MRS broth and cultured at 37 °C until stationary phase [15,16,17]. Afterwards, the bacteria were pelleted (3000× *g* for 10 min), washed twice with phosphate-buffered saline (PBS) and suspended in specific media for in vivo or in vitro assays.

Commercially available *H. pylori* ATCC43504 (American Type Culture Collection, Manassas, VA, USA) and a mouse-adapted strain *H. pylori* SS1 [19] were used for cell culture assays and to induce gastric infection in mice, respectively. Both strains were obtained from the Bacterial Pathogenicity Laboratory culture collection at University of Concepción (Concepción, Chile). Bacteria were cultured on Columbia blood agar base (Oxoid, Hampshire, UK) supplemented with 5% horse blood and antibiotics (supplement DENT, Oxoid, Hampshire, UK) in a microaerobic atmosphere (10% CO_2_, 5% O_2_, 85% N_2_) at 37 °C for 72 h. The growth form the entire plate was washed twice with PBS and suspended in PBS enriched with 5% of horse serum (GE Healthcare, Chicago, IL, USA).

### 2.2. Exopolysaccharide Extraction

The secreted EPS was isolated according to Ferrer et al. [20] with slight modifications. Briefly, a chemical-defined medium (CDM) was inoculated with (2% *v*/*v*) of an overnight culture of selected lactobacilli (grown as referred in Section 2.1) and incubated for 24 h at 37 °C under microaerobic conditions. After incubation, media were centrifuged (10,000× *g*, 30 min, at 4 °C) and the supernatant treated with trichloroacetic acid (Winkler, Chile) (final concentration 15% *v/v*) for 2 h under agitation. Then, proteins were precipitated by centrifugation (10,000× *g* for 30 min, at 4 °C) and the supernatant was mixed with 2 volumes of absolute cold ethanol (Merck, Darmstadt, Germany) followed by an overnight incubation at 4 °C, to enhance the EPS precipitation. Finally, the EPS was harvested by centrifugation (10,000× *g*, 30 min, at 4 °C) and the pellet was dissolved in distilled water (1/10 of the original volume), dialyzed at 4 °C by using dialysis membranes (14 kDa cut-off, Sigma Aldrich, St. Louis, MO, USA) and finally lyophilized [20]. For in vivo and in vitro assays, the dried EPS was suspended in PBS.

### 2.3. Cell Lines

Human gastric adenocarcinoma epithelial cells (AGS, ATCC CRL1739) were provided by the Bacterial Pathogenicity Laboratory, Department of Microbiology, University of Concepción (Concepción, Chile). Activation, propagation and freezing were carried out according to protocol for AGS cells (https://www.atcc.org/products/all/CRL-1739). AGS cells were cultured and handled according to Garcia-Castillo et al. (2018) [17]. After incubation, cells (1 × 10^5^ cells/mL) in a flat-bottom 24-well were washed with warmed PBS, and fresh culture media without antibiotics was added.

The human monocytic leukemia (THP-1) cell line was provided by the Department of Clinical Biochemistry and Immunology, University of Concepción (Concepción, Chile). Activation, propagation and freezing were carried out according to protocol for THP-1 cells (https://www.atcc.org/products/all/TIB-202). For assays, THP-1 cells were handled according to Garcia-Castillo et al. [17]. Cells were maintained in RPMI-1640 medium (Gibco) supplemented with 10% *v/v* of heat-inactivated fetal bovine serum (SFB) (Biological Industries, Cromwell, CT, USA), 100 U/mL of penicillin, and 100 μg/mL of streptomycin (Corning). THP-1 cells (1 × 10^5^ cells/mL) were incubated with 200 nM of PMA (Phorbol 12-Myristate 13-Acetate) for 24 h, to induce the differentiation of monocytes into macrophages. For immunomodulation assays, cells were washed and fresh culture media without antibiotics was added.

### 2.4. Effect of EPS on H. pylori Adhesion to AGS Cells

The effect of EPSs isolated from *L. fermentum* UCO-979C or *L. fermentum* CRL973 on the adhesion of *H. pylori* ATCC 43,504 to AGS cells was evaluated as previously described by Garcia-Castillo et al. [17] with modifications. Briefly, AGS cells were co-incubated with 100 μg/mL of UCO-979C EPS or CRL973 EPS for 24 h before the challenge with 10^7^ CFU/mL of *H. pylori* ATCC43504 for other 24 h. After incubation, AGS cells were washed and detached with 300 μL of Trypsin/EDTA 0.05% (Corning, Manassas, VA, USA), then 700 μL of DMEM without antibiotics was added. Suspension was homogenized and 10-fold dilution series were performed in PBS. Serial dilutions of the bacteria/cell suspension were seeded in selective Columbia agar plates. AGS cells infected with *H. pylori* without prophylactic treatment were considered as 100% adhesion. The UCO-979C EPS concentration was selected by preliminary experiments.

### 2.5. Effect of EPS on AGS and THP-1 Cells Cytokines Profiles

In order to evaluate the effect of EPSs on the innate immune response, AGS cells or THP-1 differentiated macrophages were incubated with 100 μg/mL UCO-979C EPS or CRL973 EPS for 24 h, and supernatants were collected for cytokines analysis. Cells without EPS stimulation were used as basal controls.

The effect of UCO-979C EPS or CRL973 EPS in the cytokine response to *H. pylori* infection was also evaluated. AGS cells or THP-1 differentiated macrophages were incubated with 100 μg/mL of UCO-979C EPS or CRL973 EPS and then challenged with 10^7^ CFU/mL of *H. pylori* ATCC43504 for 24 h. Cells without EPS stimulation and infected with *H. pylori* were used as infected controls. After incubation, supernatants were collected and kept at −80 °C to determined cytokines and chemokines by ELISA (DuoSet R&D Systems, Minneapolis, MN, USA). The levels of TNF-α, IL-6 and IL-8, and MCP-1 (pg/mL) were determined in the supernatant of AGS cells. The levels of TNF-α, IL-6, IL-10 and IFN-γ (pg/mL) were determined in the supernatant of THP-1 cells.

### 2.6. Animals and Experimental Infection

Female adult Swiss (6-week-old) SPF (specific pathogen free) mice were obtained from the closed colony kept at CERELA-CONICET (Tucuman, Argentina). They were housed in plastic cages with controlled room temperature (22 ± 2 °C temperature, 55 ± 2% humidity) and were fed ad libitum with a conventional balanced diet. Animal welfare was ensured by researchers and special trained staff in animal care and handling at CERELA-CONICET. Animal health and behavior were monitored twice a day. This study was carried out in strict accordance with the recommendations in the Guide for the Care and Use of Laboratory Animals of the Guidelines for Animal Experimentation of CERELA. The CERELA Institutional Animal Care and Use Committee prospectively approved this research under the protocol BIOT-CRL-IBT18.

Mice were treated with viable *L. fermentum* UCO-979C or *L. fermentum* CRL973 or their EPSs. Viable bacteria were administered on two consecutive days at a daily dose of 10^8^ CFU/mouse. Bacteria were suspended in 5 mL of 10% skimmed milk and added to the drinking water and the consumption was monitored ensuring each mouse drinks 5–6 mL per day. Lactobacilli viability in drinking water was assessed after 24 h by plating in MRS and Bactlight^®^ viability kit (Thermofisher, Buenos Aires, Argentina). UCO-979C EPS or CRL973 EPS were administered by drinking water at a concentration of 100 μg/mL for two consecutive days. The infection control only received water instead probiotic or EPS suspension. After two days of lactobacilli or EPSs administration, these mice and untreated controls were infected with *H. pylori* SS1 using 100 μL of 6 × 10^8^ CFU/mL of the pathogenic strain by gavage [21]. Mice were housed individually during the experiments and the assays for each parameter studied were performed in 5–6 mice per group. Two days post-infection (dpi), blood samples were collected in heparinized tubes by cardiac puncture under anesthesia. Afterwards, the stomachs were removed and processed for histological and immunological examination. To assess the gastric damage after *H. pylori* infection, stomachs were fixed in 4% (*v*/*v*) formalin saline solution, then dehydrated and embedded in paraffin wax (Leica Microsystems, Mannheim, Germany). Samples were cut in sections and stained with hematoxylin-eosin. Blind microscopic examination of slides was performed.

### 2.7. Adhesion of H. pylori to Mouse Gastric Mucosa

In order to evaluate the *H. pylori* adhesion in presence or absence of lactobacilli or EPS, stomachs were aseptically excised, weighed and dissected. Each stomach was placed in BHI broth, homogenized and diluted in PBS (10 fold). Then, dilutions were seeded on selected agar media for *H. pylori* and incubated for 72 h at 37 °C under microaerobic conditions [22]. *H. pylori* presence was confirmed by Gram staining and positive urease activity, and the concentration was expressed as CFU per gram of stomach.

Urease activity was determined in processed stomachs by a modified version of red phenol method [23,24]. Briefly, stomach samples were placed in a solution containing 0.002% of phenol red, urea 150 and 100 mM phosphate buffer and urease activity was measured by absorbance (570 nm) after 2 h by using Infinite M200 pro (TECAN) plate reader. *H. pylori* SS1-infected group mean absorbance was considered as 100% of urease activity.

### 2.8. Gastric and Serum Immunological Response

For evaluating the effect of the prophylactic administration of lactobacilli or their EPSs on the inflammatory response, cytokines and chemokines were measured. Briefly, stomachs from mice were individually grounded in a mortar with cold 5 mL of PBS and centrifuged [25,26]. Supernatant was collected and stored at −80 °C until cytokine/chemokine determination. Cytokines (IFN-γ, IL-10, TNF-α) and chemokines (IL-8, MCP-1) were quantified by enzyme-linked immunosorbent assay (ELISA) kits, following the manufacturer’s recommendations (DuoSet R&D Systems, Minneapolis, MN, USA). The values were expressed as pg per gram of stomach. In addition, to evaluate the effect of lactobacilli and EPSs treatments and infection on the systemic immune response, blood samples from mice were collected in heparinized tubes by cardiac puncture. Mouse serum samples were kept at −80 °C until cytokine/chemokine determination by ELISA kits. Values were expressed as pg/mL of serum.

### 2.9. Statistical Analysis

Experiments were performed in triplicate and results were expressed as mean ± standard deviation. After verification of the normal distribution of data, 2-way ANOVA was used. Tukey’s test (for pairwise comparisons of the means) was used to test for differences between the groups. Differences were considered significant at *p* < 0.05 or *p* < 0.01.

## 3. Results

### 3.1. L. fermentum UCO-979C Improves the Resistance Against H. pylori Infection in Mice

**Figure 1:**
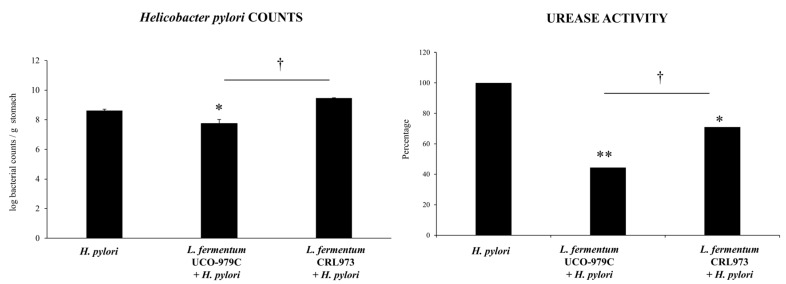
Effect of viable *L. fermentum* UCO-979C or *L. fermentum* CRL973 on gastric colonization of *H. pylori* SS1. *L. fermentum* UCO-979C or *L. fermentum* CRL973 were administered to different groups of mice for two consecutive days at a dose of 10^8^ UFC/mouse/day, then mice were challenged with *H. pylori* SS1. Two days post-infection, mice were euthanized. *H. pylori* counts (Log CFU/g of tissue) and Urease activity (% percentage) were determined in gastric explants. Mice infected with *H. pylori* were used as controls. The results represent three independent experiments and are expressed as mean ± SD. Significant differences when compared to the control group: * (*p* < 0.05),** (*p* < 0.01). Significant differences when compared to the indicated group: † (*p* < 0.05).

Our previous in vitro studies have clearly demonstrated the ability of *L. fermentum* UCO-979C to inhibit *H. pylori* growth and adhesion to human gastric epithelial cells [15] and to beneficially regulate the inflammatory response triggered by the pathogen in both epithelial cells and macrophages [17]. In this work, in a first set of experiments, we aimed to confirm the immunomodulatory and anti-*H. pylori* effects of *L. fermentum* UCO-979C in an in vivo animal model. For this purpose, adult immunocompetent mice were treated with the UCO-979 strain, as described in detail in materials and methods, and then challenged with *H. pylori*. The non-immunomodulatory strain *L. fermentum* CRL973 [18] was used for comparison. As shown in Figure 1, mice treated with *L. fermentum* UCO-979C had lower *H. pylori* colony counts associated with the stomachs, as well as reduced urease activity when compared to the control group. On the contrary, *L. fermentum* CRL973 was not able to modify the levels of *H. pylori* colonization when compared to control mice. Although the urease activity in CRL973-treated mice was lower than the observed in the *H. pylori-* infected control group, it was significantly higher when compared to that observed in UCO-979C-treated animals (Figure 1).

**Figure 2:**
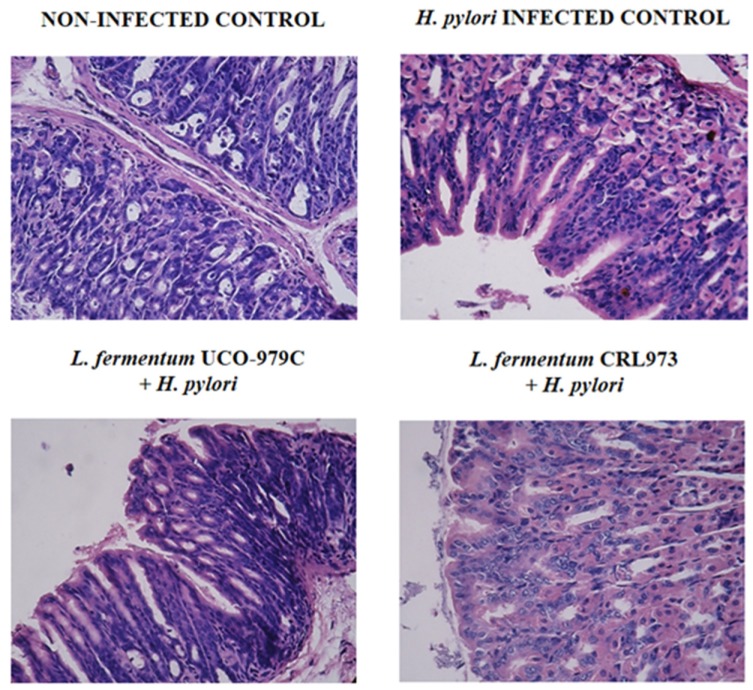
Effect of viable *L. fermentum* UCO-979C or *L. fermentum* CRL973 in the gastric inflammatory response of competent adult mice infected with *H. pylori* SS1. *L. fermentum* UCO-979C or *L. fermentum* CRL973 were administered to different groups of mice for two consecutive days at a dose of 10^8^ CFU/mouse/day, then mice were challenged with *H. pylori* SS1. Two days post-infection, mice were euthanized, and histopathological evaluation of gastric samples was performed. Hematoxilin/eosin stained sections of gastric mucosa. Upper left: Normal appearance of mice gastric mucosa. Upper right: Control group: Swiss mice infected with *H. pylori*. Lower left: *L. fermentum* UCO-979-*H. pylori* group. Lower right: *L. fermentum* CRL 973-*H. pylori* group. Original magnification 200×.

Histopathological analysis of stomach tissue sections was performed in the different groups of mice in order to evaluate the tissue damage and the inflammatory response (Figure 2). The infection of adult immunocompetent mice with *H. pylori* significantly increased the inflammatory cells infiltrating the stomach mucosa, since control mice displayed no inflammation versus notable inflammatory changes in the infected control mice. Inflammatory infiltrates consisted of neutrophils and mononuclear cells in the lamina propria and around the gastric glands, as has been described in previous publications [27,28]. The inflammation of gastric tissue in *L. fermentum* UCO-979C-treated mice was milder than that in controls after infection. There were only mild inflammatory neutrophil and mononuclear-cell infiltrates in the UCO-979C group (Figure 2). On the other hand, *L. fermentum* CRL973-treated mice showed neutrophil and mononuclear-cell infiltration in the gastric tissue that were not different from infected controls (Figure 2).

### 3.2. L. fermentum UCO-979C Differentially Modulates the Immune Response Against H. pylori Infection in Mice

**Figure 3:**
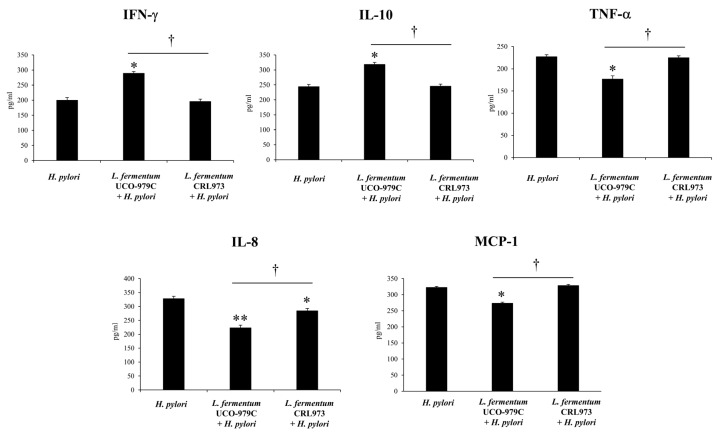
Effect of viable *L. fermentum* UCO-979C or *L. fermentum* CRL973 on gastric cytokines and chemokines *in H. pylori* SS1-infected mice. Effect of viable *L. fermentum* UCO-979C or *L. fermentum* CRL973 on gastric cytokines and chemokines in adult immunocompetent mice infected with *H. pylori* SS1. *L. fermentum* UCO-979C or *L. fermentum* CRL973 were administered to different groups of mice for two consecutive days at a dose of 10^8^ CFU/mouse/day, then mice were challenged with *H. pylori* SS1. Two days post infection, gastric concentrations of IFN-γ, IL-10, TNF-α, IL-8 and MCP-1 (pg/mL) were determined. Mice infected with *H. pylori* were used as controls. Results are expressed as mean ± SD. Significant differences when compared to the control group: * (*p* < 0.05),** (*p* < 0.01). Significant differences when compared to the indicated group: † (*p* < 0.05).

**Figure 4:**
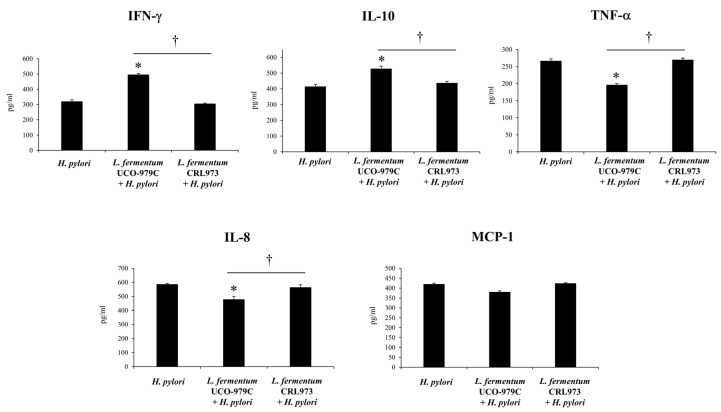
Effect of viable *L. fermentum* UCO-979C or *L. fermentum* CRL973 on serum cytokines in adult immunocompetent mice infected with *H. pylori* SS1. *L. fermentum* UCO-979C or *L. fermentum* CRL973 were administered to different groups of mice for two consecutive days at a dose of 10^8^ CFU/mouse/day, then mice were challenged with *H. pylori* SS1. Two days post-infection, serum concentrations of IFN-γ, IL-10, TNF-α, IL-8 and MCP-1 (pg/mL) were determined. Mice infected with *H. pylori* were used as controls. Results are expressed as mean ± SD. Significant differences when compared to the control group: * (*p* < 0.05). Significant differences when compared to the indicated group: † (*p* < 0.05).

Cytokines and chemokines were measured in the stomach (Figure 3) and serum (Figure 4) samples of lactobacilli-treated and untreated control groups after the challenge with *H. pylori*. The infection of adult immunocompetent mice with *H. pylori* significantly increased the levels of the inflammatory factors IFN-γ, TNF-α, MCP-1 and IL-8, as well as the levels of the regulatory cytokine IL-10 when compared to basal levels in non-infected animals (data not shown). Mice treated with *L. fermentum* UCO-979C had significantly higher levels of gastric and serum IFN-γ when compared to the control group (Figure 3 and Figure 4). On the contrary, the UCO-979C-treated group showed significantly lower levels of gastric and serum TNF-α and IL-8 compared to the control group (Figure 3 and Figure 4). *L. fermentum* UCO-979C was also able to reduce the levels of MCP-1 in the gastric mucosa when compared to controls (Figure 3). In addition to its ability to diminish inflammatory cytokines and chemokines, it was observed that the UCO-979C strain was capable of inducing a significant increase in IL-10 levels when compared to controls in both the gastric mucosa (Figure 3) and serum (Figure 4). The group of animals treated with *L. fermentum* CRL973 did not exhibit any variation in gastric IFN-γ, IL-10, TNF-α, MCP-1 levels and only gastric IL-8 values were lower than that observed in the control group (Figure 3). On serum samples, there were not statistically significant differences between *L. fermentum* CRL973 and control mice (Figure 4).

### 3.3. The EPS from L. Fermentum UCO-979C Reduces H. pylori Adhesion to AGS Cells

**Figure 5:**
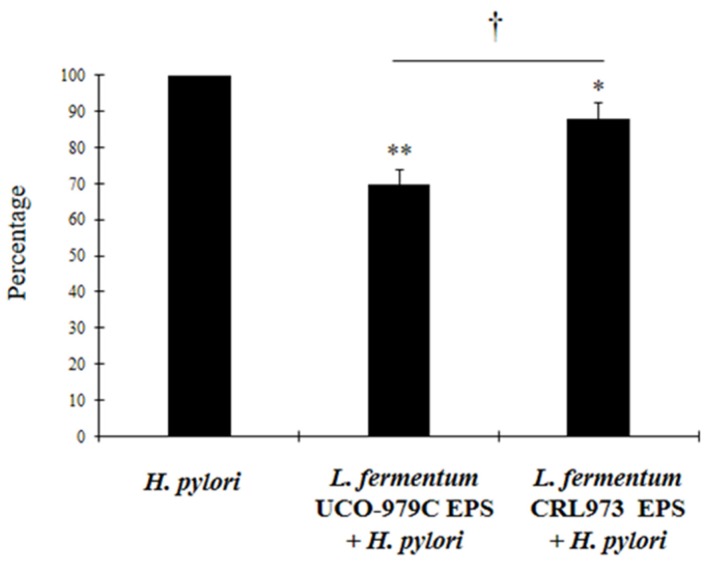
Effect of *Lactobacillus fermentum* UCO-979C EPS pre-incubation on *Helicobacter pylori* adhesion to human gastric epithelial cells (AGS cells) challenged with *H. pylori.* AGS cells were stimulated with EPS-979C or EPS-973 for 24 h before the challenge. Results are expressed in percentage, 100% correspond to infected control and represent data from three independent experiments. Results are expressed as mean ± SD. Significant differences when compared to the control group: * (*p* < 0.05), ** (*p* < 0.01). Significant differences when compared to the indicated group: † (*p* < 0.05).

In a second set of experiments, we evaluated whether the EPS produced by *L. fermentum* strain UCO-979C was involved in the in vitro and in vivo abilities to improve resistance against *H. pylori* and differentially modulate the immune response. We first evaluated the ability of the EPS produced by the UCO-979C to modify the adhesion of *H. pylori* to AGS cells. For this purpose, AGS cells were treated with UCO-979C EPS prior to *H. pylori* infection. The EPS isolated from strain *L. fermentum* CRL973 was used for comparison. As shown in Figure 5, both UCO-979C EPS and CRL973 EPS significantly reduced *H. pylori* adherence. However, the protective effect of UCO-979C EPS was greater than that for CRL973 EPS (Figure 5).

### 3.4. The EPS from L. fermentum UCO-979C Modulates Cytokine Profile in AGS and THP-1 Cells

**Figure 6:**
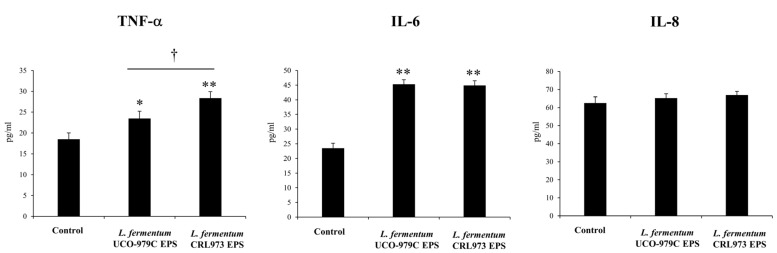
Effect of *Lactobacillus fermentum* UCO-979C or *L. fermentum* CRL973 EPS on cytokine and chemokine production of human gastric epithelial cells (AGS cells). AGS cells were incubated with 100 μg/mL of *L. fermentum* UCO-979C or *L. fermentum* CRL973 EPS. The levels of TNF-α, IL-6 and IL-8 (pg/mL) in culture supernatants were determined 24 h after stimulation. The results represent data from three independent experiments. Results are expressed as mean ± standard deviation. Significant differences when compared to the control group: * (*p* < 0.05), ** (*p* < 0.01). Significant differences when compared to the indicated group: † (*p* < 0.05).

We next aimed to evaluate whether the EPS produced by the probiotic strain *L. fermentum* UCO-979C was able to modify the production of cytokines and chemokines in human gastric epithelial cells and macrophages. AGS cells were stimulated with UCO-979C EPS and the levels of TNF-α, IL-6 and IL-8 were measured in culture supernatants (Figure 6). Again, the EPS isolated from *L. fermentum* CRL973 was used for comparison. The levels of TNF-α and IL-6 were significantly increased in the presence of UCO-979C EPS or CRL973 EPS. However, CRL973 EPS was more efficient than UCO-979C EPS to enhance TNF-α production in epithelial cells. On the contrary, IL-8 levels were not affected by UCO-979C EPS or CRL973 EPS treatments (Figure 6).

**Figure 7:**
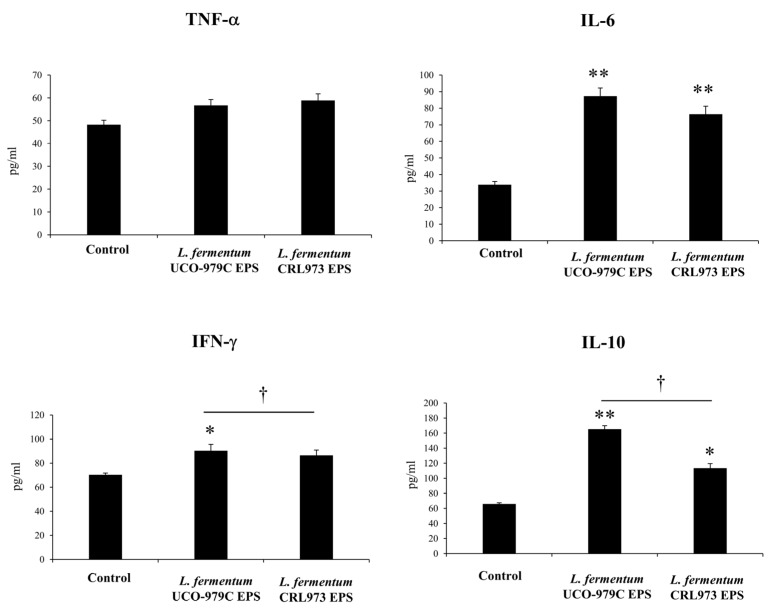
Effect of *Lactobacillus fermentum* UCO-979C or *L. fermentum* CRL973 EPS on cytokine and chemokine production of human macrophages (THP-1 cells). THP-1 cells were incubated with 100 μg/mL of *L. fermentum* UCO-979C or *L. fermentum* CRL973 EPS. The levels of TNF-α, IL-6, IFN-γ and IL-10 (pg/mL) in culture supernatants were determined 24 h after stimulation. The results represent data from three independent experiments. Results are expressed as mean ± standard deviation. Significant differences when compared to the control group: * (*p* < 0.05), ** (*p* < 0.01). Significant differences when compared to the indicated group: † (*p* < 0.05).

When the effect of EPSs on THP-1 cells was analyzed, it was observed that both UCO-979C EPS and CRL973 EPS significantly increased the levels of IL-6 and IL-10 (Figure 7). However, UCO-979C EPS was more efficient than CRL973 EPS to enhance IL-10 production in macrophages. The levels of TNF-α were not affected by UCO-979C EPS or CRL973 EPS treatments, while only the EPS produced by the probiotic strain *L. fermentum* UCO-979C was able to increase the production of IFN-γ (Figure 7).

**Figure 8:**
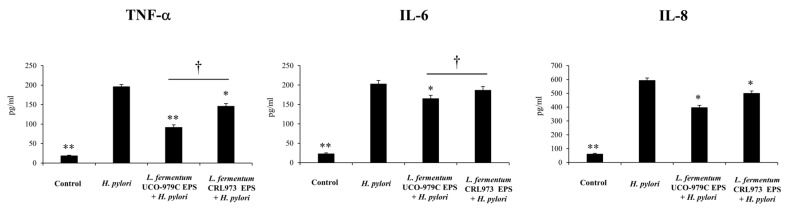
Effect of *Lactobacillus fermentum* UCO-979C or *L. fermentum* CRL973 EPS on the cytokine and chemokine production of human gastric epithelial cells (AGS cells) after *Helicobacter pylori* challenge. AGS cells were incubated with 100 μg/mL of *L. fermentum* UCO-979C or *L. fermentum* CRL973 EPS for 24 h. Then, cells were challenged with *H. pylori* 43504. The levels of TNF-α, IL-6, IFN-γ and IL-10 (pg/mL) in culture supernatants were determined 24 h after infection. Results represent data from three independent experiments. Results are expressed as mean ± standard deviation. Significant differences when compared to the control group: * (*p* < 0.05), ** (*p* < 0.01). Significant differences when compared to the indicated group: † (*p* < 0.05).

**Figure 9:**
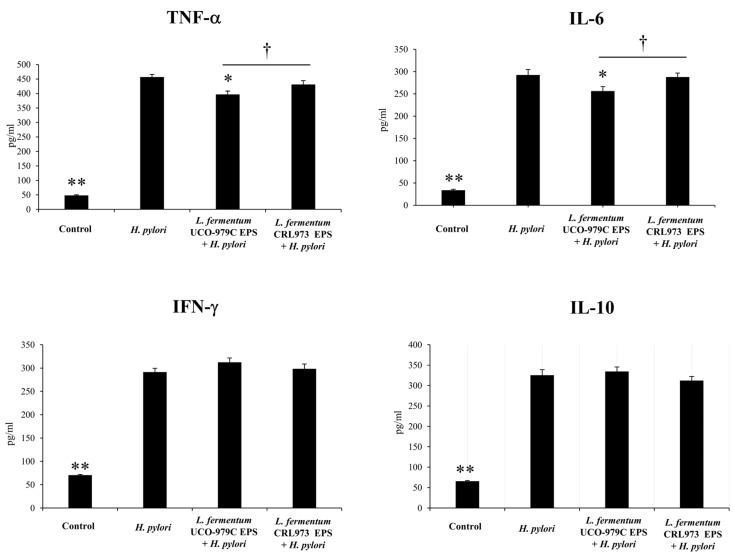
Effect of *Lactobacillus fermentum* UCO-979C or *L. fermentum* CRL973 EPS on cytokine and chemokine production of human macrophages (THP-1 cells) after *Helicobacter pylori* challenge. THP-1 cells were incubated with 100 μg/mL of *L. fermentum* UCO-979C or *L. fermentum* CRL973 EPS for 24 h. Then, cells were challenged with *H. pylori* 43504. The levels of TNF-α, IL-6, IFN-γ and IL-10 (pg/mL) in culture supernatants were determined 24 h after infection. The results represent data from three independent experiments. Results are expressed as mean ± standard deviation. Significant differences when compared to the control group: * (*p* < 0.05), ** (*p* < 0.01). Significant differences when compared to the indicated group: † (*p* < 0.05).

We also evaluated whether the EPS of *L. fermentum* UCO-979C was able to modify the production of cytokines and chemokines in human gastric epithelial cells and macrophages in the context of *H. pylori* infection. It was observed that after the infection with *H. pylori*, secretion of TNF-α, IL-6 and IL-8 increased considerably in AGS cells (Figure 8) compared to basal levels (Figure 6). Likewise, TNF-α, IL-6, IFN-γ and IL-10 significantly increased in THP-1 cells (Figure 9) when compared to basal levels (Figure 7).

AGS cells stimulated with UCO-979C EPS showed a significant decrease in the levels of the inflammatory cytokines TNF-α, IL-6 and IL-8 when compared with *H. pylori*-infected controls (Figure 8). In addition, it was observed that the levels of TNF-α and IL-8 decreased significantly in AGS cell treated with CRL973 EPS than in the control group (Figure 8). However, it should be noted that TNF-α was significantly lower in UCO-979C EPS-treated AGS cells than in those stimulated with CRL973 EPS.

On the other hand, the treatment of THP-1 cells with UCO-979C EPS previous to infection with *H. pylori* did not exert any effect on the levels of IFN-γ and IL-10, since the concentrations of both cytokines were not different from the control infected THP-1 cells (Figure 9). In addition, a modest but significant decrease in the levels of TNF-α and IL-6 was observed in THP-1 cells treated with UCO-979C EPS when compared to the infected controls (Figure 9). There were not statistical differences in the levels of TNF-α, IL-6, IFN-γ or IL-10 when cells treated with the EPS from *L. fermentum* CRL973 and infected control cells were compared (Figure 9).

### 3.5. The EPS from L. fermentum UCO-979C Modulates the Inflammatory Response Triggered by H. pylori Infection in Mice

**Figure 10:**
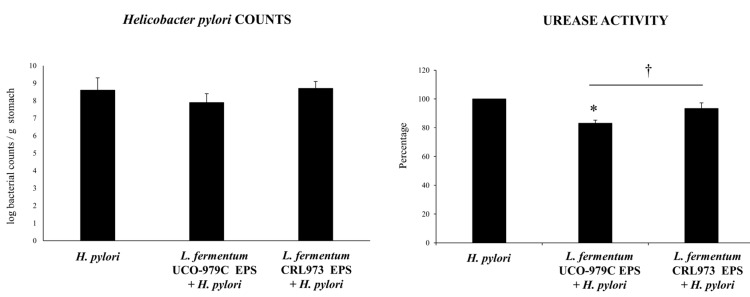
Effect of *L. fermentum* UCO-979C or *L. fermentum* CRL973 EPS on gastric colonization of *H. pylori* SS1. *L. fermentum* UCO-979C or *L. fermentum* CRL973 EPS were administered to different groups of mice for two consecutive days at a dose of 10^8^ 100μg/mL/mouse/day, then mice were challenged with *H. pylori* SS1. Two days post-infection, mice were euthanized, *H. pylori* counts (Log CFU/g of tissue) and Urease activity (% percentage) were determined in gastric explants. Mice infected with *H. pylori* were used as controls. Results are expressed as mean ± standard deviation. Significant differences when compared to the control group: * (*p* < 0.05). Significant differences when compared to the indicated group: † (*p* < 0.05).

Finally, we aimed to determine whether the EPS from *L. fermentum* UCO-979C was able to exert beneficial effects in vivo. For this purpose, mice were orally treated with an aqueous suspension of the lyophilized UCO-979C EPS and then challenged with *H. pylori*. Mice treated with CRL973 EPS were used for comparisons. As shown in Figure 10, it was observed that the treatment of mice with UCO-979C EPS did not induce significant differences in the counting of pathogenic bacteria in stomach samples. However, a significant reduction in the urease activity in UCO-979C EPS mice was detected when compared to controls (Figure 10). As expected, there were not statistical differences in *H. pylori* counts or urease activity when mice treated with the EPS from *L. fermentum* CRL973 and infected controls were compared (Figure 10).

**Figure 11:**
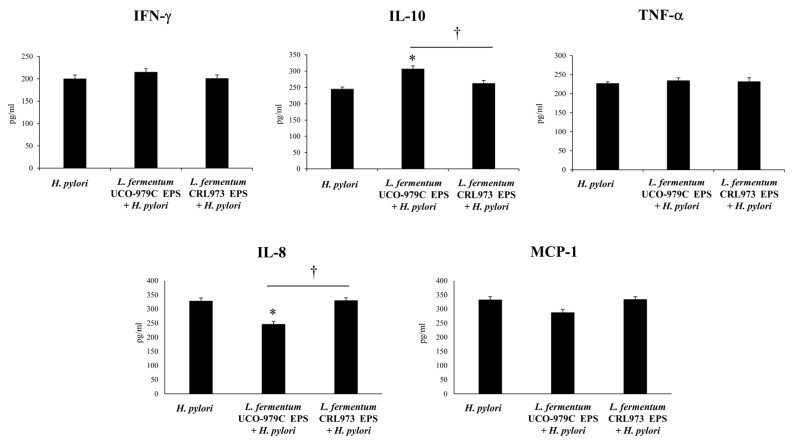
Effect of *L. fermentum* UCO-979C or *L. fermentum* CRL973 EPS on gastric cytokines and chemokines in adult immunocompetent mice infected with *H. pylori* SS1. *L. fermentum* UCO-979C or *L. fermentum* CRL973 EPS were administered to different groups of mice for two consecutive days at a dose of 100μg/mL mouse/day, then mice were challenged with *H. pylori* SS1. Two days post-infection, gastric concentrations of IFN-γ, IL-10, TNF-α, IL-8 and MCP-1 (pg/mL) were determined. Mice infected with *H. pylori* were used as controls. Results are expressed as mean ± standard deviation. Significant differences when compared to the control group: * (*p* < 0.05). Significant differences when compared to the indicated group: † (*p* < 0.05).

**Figure 12:**
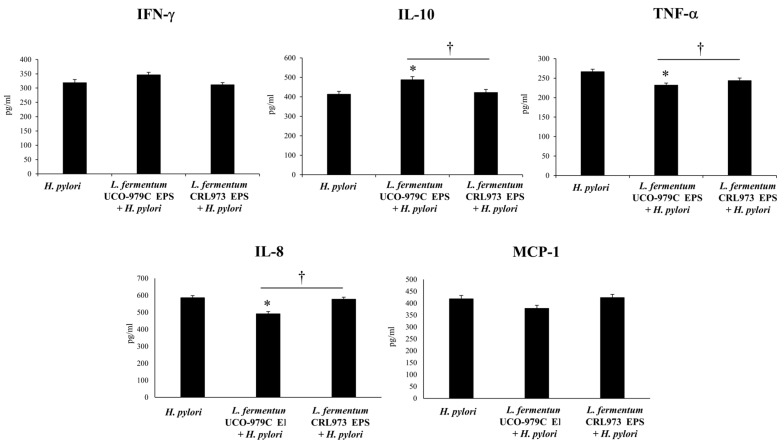
Effect of *L. fermentum* UCO-979C or *L. fermentum* CRL973 EPS on serum cytokines and chemokines in adult immunocompetent mice infected with *H. pylori* SS1. *L. fermentum* UCO-979C or *L. fermentum* CRL973 EPS were administered to different groups of mice for two consecutive days at a dose of 100 μg/mL mouse/day, then mice were challenged with *H. pylori* SS1. Two days post-infection, serum concentrations of IFN-γ, IL-10, TNF-α, IL-8 and MCP-1 (pg/mL) were determined. Mice infected with *H. pylori* were used as controls. Results are expressed as mean ± standard deviation. Significant differences when compared to the control group: * (*p* < 0.05). Significant differences when compared to the indicated group: † (*p* < 0.05).

In order to determine the impact of UCO-979C EPS on the innate immune response, we measured gastric (Figure 11) and systemic (Figure 12) cytokines and chemokines in infected mice. It was observed that the administration of UCO-979C EPS was capable of reducing the levels of serum and gastric IL-8 as well as serum TNF-α in *H. pylori*-infected mice. In addition, UCO-979C EPS-treated mice had significantly higher levels of gastric (Figure 11) and serum (Figure 12) IL-10 than infected controls. No differences were detected in the levels of IFN-γ and MCP-1 when UCO-979C EPS and control mice were compared. The CRL973 EPS did not exert any significant effects in the levels of gastric or serum cytokines and chemokines during *H. pylori* infection (Figure 11 and Figure 12).

## 4. Discussion

*H. pylori* triggers mucosal and systemic immune responses in infected individuals. However, these immune responses are not always efficient for eradicating the pathogen [29,30]. Moreover, the aggressive and persistent proinflammatory response induced by this pathogen can contribute to the development of gastritis, preceding a series of morphological changes that may lead to gastric cancer. An enhanced modification in DNA methylation in the gastric mucosa, which is considered as a preliminary stage of tumor transformation, has been reported after *H. pylori* infection [31]. Moreover, the infection-associated inflammatory response rather than *H. pylori* itself has been associated to DNA methylation [31,32]. Then, mediators of inflammation including cytokines and chemokines, are considered as important therapeutic targets to prevent gastritis and gastric cancer associated to *H. pylori* infection [33]. In this regard, the beneficial effect of probiotics for the protection against *H. pylori* infection and inflammation is supported by a large amount of scientific evidence [13,14,34]. Earlier studies reported that *L. salivarius* WB 1004 [35], *L. rhamnosus* R0011 and *L. acidophilus* R0052 [36] were able to differentially modulate immune responses and diminish *H. pylori* colonization in mice. The beneficial modulation of the inflammatory immune response and the reduction of *H. pylori* adhesion has been also reported in in vitro experiments for probiotic strains such as *L. bulgaricus* [37] and *L. rhamnosus* UCO-25A [38]. Moreover, studies have proposed the inactivation of Smad7 and NF-κB signaling pathways [39] and the activation of SOCS-2/SOCS-3 signaling through STAT1/STAT3 activation and JAK2 inactivation [40] as the molecular mechanisms of action for the beneficial effects of probiotics. Then, these and other in vitro and in vivo studies have clearly demonstrated the potential of probiotics for the protection against *H. pylori* colonization as well as in the regulation of the associated inflammation.

We have carried out studies to evaluate the ability of probiotics to protect against *H. pylori* infection using mainly the strain *L. fermentum* UCO-979C. This probiotic strain was selected between other of lactic acid bacteria isolated from human gastric tissue because of its remarkable anti-*H. pylori* properties [41]. The UCO-979C strain strongly inhibited the adhesion, growth and urease activity of *H. pylori* in AGS cells and Mongolian gerbils [16,42]. Moreover, *L. fermentum* UCO-979C was able to beneficially modulate the cytokine response of AGS cells and THP-1 macrophages after *H. pylori* challenge [17]. Here, we extend those previous findings by demonstrating, for the first time, the ability of *L. fermentum* UCO-979C to beneficially modulate the innate immune response triggered by *H. pylori* in vivo.

Cytokines variations during the *H. pylori* infection have a significant impact on the evolution of the gastric pathology due to their vast and pleiothropic effects on immune and epithelial cells [43]. The increased production of proinflammatory cytokines and chemokines, including TNF-α, IL-6, IL-8 and MCP-1, during *H. pylori* gastric mucosal inflammation, has been well documented. Those inflammatory factors can be secreted by gastric epithelial cells and play a major role in triggering the mucosal inflammatory damage caused by *H. pylori* [44,45]. In addition, *H. pylori* and its virulence factors are capable of increasing the production of the inflammatory mediators TNF-α, IL-6, and IFN-γ by macrophages, which contribute to the amplification of the inflammatory response in the gastric mucosa [46,47]. In our previous study [17], we demonstrated that *L. fermentum* UCO-979C significantly reduced the production of IL-8, TNF-α, IL-6, and MCP-1 in AGS cells and macrophages challenged with *H. pylori*. The results presented here confirm those in vitro findings, by demonstrating that the UCO-979C strain reduced the levels of TNF-α, IL-8, and MCP-1 in the gastric mucosa of *H. pylori*-infected mice. In addition, our previous in vitro studies also revealed the ability of the UCO-979C strain to improve the levels of IL-10 in *H. pylori*-infected macrophages [17], which is in line with the increased concentrations of IL-10 found in gastric and serum samples of *H. pylori*-infected mice. Increased levels of IL-10 may contribute to the chronicity of gastritis, however, this regulatory cytokine is of fundamental importance to prevent mucosal injury mediated by the inflammatory response [47,48,49,50]. Then, the balance in the inflammatory and regulatory cytokine production induced by *L. fermentum* UCO-979C could offer advantages in the protection against *H. pylori* infection, since a reduced inflammatory damage was observed in the histopathological analysis of gastric samples of mice.

The differential cytokine profile induced by *L. fermentum* UCO-979C treatment in *H. pylori*-infected mice could also explain the reduction in the pathogen gastric colonization. The generation of a Th1 response with the subsequent increase in the mucosal and systemic levels of IFN-γ have been described in both experimental animal models and human clinical trials [51,52]. The production of appropriate levels of IFN-γ has been associated to the protection against *H. pylori* infection. Peek et al. [53] demonstrated that mice that are deficient in IFN-γ have an increased susceptibility to *H. pylori* colonization. It was also reported that the virulence factor cytotoxin-associated gene A (cagA) can be translocated into the cytoplasm of dendritic cells, reducing the secretion of IL-12p40 and impairing the generation of the Th1 response, which would favor the replication of *H. pylori* [54,55]. Then, the increase in gastric IFN-γ induced by probiotics such as *L. fermentum* UCO-979C could reduce the initial replication of *H. pylori* in the initial steps of the infection.

Some studies have demonstrated that probiotic strains may exert beneficial effects on *H. pylori* infection and inflammation through the molecules produced and secreted by bacterial cells [56]. Interestingly, it was reported that *L. rhamnosus* GG-conditioned media was able to antagonize TNF-α secretion induced by *H. pylori* or LPS in murine macrophages [57]. In addition, the supernatant of *L. plantarum* B7 administered to rats infected with *H. pylori* showed the ability to reduce gastric pathology and apoptotic cells rate, as well as to decrease serum TNF-α and MDA levels [58]. Among the functional molecules produced by probiotics, are EPSs macromolecules, which were proposed to be involved in host–microbe interactions [59]. In that regard, a polysaccharide produced by *Bacteroides fragilis* can suppress IL-17 and increase IL-10 production by intestinal immune cells and in a rodent model of *Helicobacter hepaticus* infection [60]. In addition, it was described that polysaccharides produced by *L. salivarius* B37 and *L. salivarius* B60 suppressed *H. pylori*-induced IL-8 production and mRNA expression in gastric epithelial cells [61]. To our knowledge, no other studies have demonstrated the anti-*H. pylori* inhibitory and immunomodulatory effects of EPS obtained from a probiotic strain.

We have reported that *L. fermentum* UCO-979C produces large amount of EPS and is able to form biofilms on AGS and Caco-2 cell lines, inhibiting the colonization by *H. pylori* by up to 80% [15]. Thus, it was concluded that the EPS was a key molecule in the ability of *L. fermentum* UCO-979C to inhibit *H. pylori* colonization. In this work, we hypothesized that UCO-979C EPS is also involved in the immunomodulatory effects of this probiotic strains in the context of *H. pylori* infection. We confirmed that the UCO-979C EPS was capable of inducing a significant reduction in *H. pylori* adhesion (~30%) to AGS cells, which was comparable with the inhibitory activity of viable *L. fermentum* UCO-979C (~44%) reported previously [17]. In addition, the UCO-979C EPS was able to differentially modulate the cytokine profile of AGS and THP-1 cells in response to the challenge with *H. pylori*. Similar to our previous studies with *L. fermentum* UCO-979C [17], the UCO-979C EPS reduced the production of TNF-α, IL-6 and IL-8 in *H. pylori*-infected AGS cells while it diminished the production of TNF-α in *H. pylori*-infected THP-1 cells. Moreover, the in vivo studies of this work demonstrated that both *L. fermentum* UCO-979C and its EPS were able to reduce IL-8 and enhance IL-10 levels in the gastric mucosa of infected mice. It should be noted, however, that the immunological changes induced by UCO-979C EPS in our in vitro and in vivo experiments did not completely resemble those observed for the viable UCO-979C strain. The UCO-979C EPS was not able to improve the levels of IFN-γ in infected mice, as observed for the viable bacterium. The lack of ability of UCO-979C EPS to increase IFN-γ could be related to its inability to reduce *H. pylori* counts in vivo. In this sense, it would be of great value to evaluate alternative treatments with UCO-979C EPS, varying concentrations and periods of administration, to conclusively rule out its ability to increase gastric IFN-γ in vivo.

The molecular mechanisms by which *L. fermentum* UCO-979C or its EPS modulate the secretion of inflammatory cytokines and chemokines in *H. pylori*-infected AGS cells are not known. Some studies have described the ability of probiotic lactobacilli to differentially modulate the expression of inflammatory factors in gastric epithelial cells through the suppression of NF-κB activation [39,62]. Recently, we described the ability of *L. fermentum* UCO-979C to modify the expression of negative regulators of TLR4 signaling in intestinal epithelial cells [18]. The UCO-979C strain diminished the expression of MKP-1 and Tollip in intestinal epithelial cells after the activation of TLR4, conducting the down-regulation in the expression of inflammatory factors such as *IL-8*, *CXCL9*, *CXCL10*, *CXCL11*, and *C3*. Then, it is tempting to speculate that *L. fermentum* UCO-979C or its EPS are able to interact with pattern recognition receptors expressed in gastric epithelial cells, inducing the upregulation of negative regulators and thereby modifying the activation of signaling pathways such as the NF-κB pathway, and reducing the production of inflammatory mediators. The detailed molecular investigation of this hypothesis is an interesting topic for future research.

In order to evaluate whether the immunomodulatory effects of *L. fermentum* UCO-979C or its EPS in the context of *H. pylori* infection were a strain-specific property, the in vitro and in vivo experiments conducted in this work were performed in comparison with a strain of the same species, which is also able to produce EPS. Although CRL973 EPS was able to slightly reduce *H. pylori* adhesion to AGS cells and diminish the production of IL-8 and TNF-α by the gastric epithelial cells in vitro, neither the CRL973 strain nor its EPS were able to induce changes in *H. pylori* colonization or protect against inflammatory damage in infected mice. Interestingly, CRL973 EPS was not able to induce any significant effect in the cytokine profiles of THP-1 cells or gastric samples of infected mice. These results allow us to arrive at two conclusions. First, the modulation of immune cells (such as macrophages) would be of great relevance to achieve an optimal protective effect in vivo. This implies that the in vitro selection and characterization of immunomodulatory bacteria or their functional molecules for their application in the prevention of *H. pylori* infection should not only be limited to the use of epithelial cells but also include specialized cells of the immune system. Secondly, our results indicate that the EPS produced by *L. fermentum* UCO-979C would have unique functional characteristics that deserve to be studied in depth. Chemical, structural, genetic and genomic studies comparing the EPS of the UCO-979C and CRL973 strains as well as their ability to interact with different pattern recognition receptors would be of great importance to advance the knowledge of the molecular mechanisms involved in the effect of beneficial microorganisms against *H. pylori* infection.

The stomach mucosa is protected against pathogenic microbes by the low gastric pH, the secretion of antimicrobial peptides and mucins by epithelial cells [63], as well as by the presence of an associated microbiota [64]. Disbyosis of gastric microbiota and inefficient production of antimicrobial peptides such as β-defensins have been implicated in an increased susceptibility to *H. pylori* infection [63,64]. Taking into consideration that the probiotic strain *L. fermentum* UCO-979C was originally isolated from the healthy human gastric mucosa, it would be of value to investigate its influence on the gastric microbiota, the production of antimicrobial peptides as well as the interaction among them, to further characterize the mechanisms involved in its beneficial effects.

In conclusion, this work demonstrates for the first time the in vivo ability of the probiotic strain *L. fermentum* UCO-979C to improve the resistance against *H. pylori* infection by modulating the gastric innate immune response. In addition, our results demonstrate that the EPS expressed by *L. fermentum* UCO-979C is partially responsible for its immunomodulatory effect, impacting its anti-inflammatory activity.
